# Alteration of *TAC1* expression in *Prunus* species leads to pleiotropic shoot phenotypes

**DOI:** 10.1038/s41438-018-0034-1

**Published:** 2018-05-01

**Authors:** Courtney A. Hollender, Jessica M. Waite, Amy Tabb, Doug Raines, Srinivasan Chinnithambi, Chris Dardick

**Affiliations:** 1 USDA-ARS Appalachian Fruit Research Station, Kearneysville, WV 25430 USA; 2 Present Address: Department of Horticulture, Michigan State University, East Lansing, MI 48824 USA

## Abstract

*Prunus persica* (peach) trees carrying the “Pillar” or “Broomy” trait (*br*) have vertically oriented branches caused by loss-of-function mutations in a gene called *TILLER ANGLE CONTROL 1* (*TAC1*). *TAC1* encodes a protein in the IGT gene family that includes *LAZY1* and *DEEPER ROOTING 1* (*DRO1*), which regulate lateral branch and root orientations, respectively. Here we found that some of the native *TAC1* alleles in the hexaploid plum species *Prunus domestica*, which has a naturally more upright stature, contained a variable length trinucleotide repeat within the same exon 3 region previously found to be disrupted in pillar peach trees. RNAi silencing of *TAC1* in plum resulted in trees with severely vertical branch orientations similar to those in pillar peaches but with an even narrower profile. In contrast, *PpeTAC1* overexpression in plum led to trees with wider branch angles and more horizontal branch orientations. Pillar peach trees and transgenic plum lines exhibited pleiotropic phenotypes, including differences in trunk and branch diameter, stem growth, and twisting branch phenotypes. Expression profiling of pillar peach trees revealed differential expression of numerous genes associated with biotic and abiotic stress, hormone responses, plastids, reactive oxygen, secondary, and cell wall metabolism. Collectively, the data provide important clues for understanding *TAC1* function and show that alteration of *TAC1* expression may have broad applicability to agricultural and ornamental tree industries.

## Fruit trees: Slimmer trees for denser planting

Peach and plum trees grow tall and slim when the *TAC1* gene is mutated, permitting higher-density planting in orchards. Branches of peach trees with the “pillar” trait grow more “up” than “out”, reducing each tree’s footprint. Chris Dardick at the Appalachian Fruit Research Station in West Virginia and co-workers previously traced the trait to mutation of *TAC1*. However, the effects of *TAC1* mutation on tree shape in other species remains poorly understood. Dardick and co-workers investigated the effects of knocking out *TAC1* in peach and plum trees. In both species, trees were narrower, but fruit yield was unchanged. Both species also showed other effects, including differences in trunk and branch thickness; these effects differed between peach and plum. These data may help in breeding of more upright fruit trees, improving fruit yield per acre.

## Introduction

Given the limited availability of agricultural land and water, increases in crop productivity will require higher-density agricultural production to keep pace with the growing worldwide demand for food. During key stages of the domestication of cereal crops such as rice and maize, considerable gains in productivity were achieved through modifications in plant architecture. This included reduced tillering and breeding for upright tiller and leaf angles, which minimizes competition and increases the efficiency of light capture under crowded conditions^1,2^. There is a growing body of evidence that pillar or columnar shapes have the potential to increase crop productivity in fruit trees as they have for cereal crops. The “Broomy” (*br*) trait in peach was previously bred to produce commercial quality peach and nectarine varieties with a pillar or, in the case of heterozygous individuals, an upright stature^3–5^. The narrower canopy of these trees was shown to improve dry matter partitioning due to more effective light interception when the angle of the sun is less than vertical^6,7^. In addition to branch angle, pleotropic phenotypes have also been described in pillar peach trees, including fewer sylleptic branches, shorter branches, longer internodes, and increased auxin and auxin-to-cytokinin ratios, features possibly associated with increased apical dominance^8^. A similar trait in apple called columnar or *Co* is also being bred for integration into high-density orchard systems^9^. *Co* trees also have narrow branch angles, fewer sylleptic shoots, and higher apical dominance^10^. But unlike pillar peaches, columnar apples produce numerous short spurs and have thickened stems^10^. The *Co* locus has been shown to contain a *Ty3*-type transposable element that is not inserted within a known gene but may act, in part, through altered expression of a neighboring 2-oxoglutarate (2OG)–Fe(II) oxygenase^11,12^. The ultimate utility of pillar or columnar traits to improve productivity in orchards will require a greater understanding of how the underlying genes function and their roles in overall plant development.

To date, there is relatively little known about the genes and molecular mechanisms that contribute to changes in tree branch orientation and much of what is known comes from work in monocots. Three genes were previously identified in cereal crops that are associated with upright tiller and/or leaf angle, *TILLER ANGLE CONTROL 1* (*TAC1*) and two zinc finger proteins *LOOSE PLANT ARCHITECTURE* 1 (*LPA1*) and *PROSTRATE GROWTH 1* (*PROG1*)^2,13,14^. *LPA1* and its Arabidopsis ortholog *SHOOT GRAVITROPISM 5* (*SGR5*) regulate shoot gravitropism upstream of amyloplast sedimentation^14,15^. No orthologs of rice *PROG1* have yet been identified in dicots.

We previously showed that pillar traits in peach are caused by mutations within a *TAC1* ortholog^16^. Changes in *TAC1* have since been linked to upright tiller or branch angles in other plant species including Arabidopsis, *Miscanthus sinensis*,* Brassica napus* L. (rapeseed) and *Populus* species (poplar)^17–19^. *TAC1* was found to be expressed to varying degrees in shoot tips, branch nodes, leaf axils, and flower buds in Arabidopsis, peach, and poplar^13,16,19,20^. In field-grown peach trees, *TAC1* expression was seasonal, being highest in early spring and coinciding with lower auxin levels^21^.

In most plant genomes, *TAC1* typically occurs as a single gene and putative TAC1 proteins contain no known domains or motifs nor have any molecular functions been ascribed to date^16^. However, *TAC1* shares distant sequence similarity with other known plant architecture regulators including *LAZY1* and *DEEPER ROOTING 1* (*DRO1*), which together comprise a larger gene family dubbed IGT for a conserved amino acid triplet found within domain II of the protein^16,22,23^. In rice and Arabidopsis, *lazy1* mutants display the opposite phenotype as *tac1*, having wider lateral tillers or branches, while *dro1* mutants have wider lateral root angles^23–28^. In Arabidopsis, DRO family members were recently shown to also play a minor role in contributing to shoot architecture^28,29^. *LAZY1* mutant phenotypes are associated with reduced gravitropic responses. These proteins appear to function downstream of statolith sedimentation but upstream of auxin translocation^24–26,28–30^, though no specific molecular or cellular functions are known at this time. The primary difference between TAC1 and LAZY/DRO proteins is the presence of a C-terminal domain (domain V) containing a putative Ethylene-responsive element binding factor-associated Amphiphilic Repression motif, which is not present in TAC1 proteins^16,23,28^. A role for *TAC1* in regulating gravitropism has not been established; however, based on the phenotypic, phylogenetic, and expression patterns of *TAC1* and *LAZY1*, we hypothesized that *TAC1* may alter shoot growth angle by negatively regulating *LAZY1*^16^.

Here we further characterized *TAC1* in *Prunus* species to better understand both the functional role of this gene and the potential applicability to producing upright fruit trees for high-density orchard systems.

## Materials and methods

### Peach and plum germplasm

The peach population used for structural measurements and expression profiling was derived from a self-pollinated tree heterozygous for the pillar trait (KV991636) in 2002. KV991636 was a seedling from a cross between Kv93065 (a pillar tree) as the female parent and pollen from the peach variety “Weeping White”. Leaf samples from the plum variety “Improved French” were used for Illumina genome sequencing. For plum transformation and associated controls, seedlings derived from open-pollinated “Bluebyrd” or “President” trees were used.

### Plum Illumina sequencing

Genomic DNA was extracted from leaves of “Improved French” plum trees as previously described^16^. Briefly, leaves were pulverized in liquid nitrogen and genomic DNA was extracted using the EZNA^TM^ High Performance (HP) DNA Kit (Omega Bio-Tek Inc., http://www.omegabiotek.com) with the addition of 2% polyvinylpyrrolidone-40 (PVP-40) (w/v) to CPL buffer and 2-mercaptoethanol. Genomic DNA quantity was assessed using the Quant-iT PicoGreen Kit (Invitrogen, Carlsbad, CA).

Two micrograms of purified DNA was provided to David H Murdock Research Institute, Kannapolis, NC for library construction and sequencing. Both a paired-end and a mate-pair library were constructed with average insert sizes of 375 bp and 2950, respectively, and sequenced using an Illumina HiSeq 2000. A total of 194,856,870 100 bp paired-end reads and 158,319,386 mate-pair reads were obtained. Reads were assembled against a 5 kb *TAC1* genomic region (Chr#2: 19,657,195–19,662,152) (peach genome version 1) using the CLC Genomics Workbench (Qiagen, USA) reference assembly tool with 2 modifications to the default settings: length fraction = 0.7, similarity fraction = 0.9^31^. The *TAC1* genic region had an average coverage of ~100 reads per base representing 14× coverage of the hexaploid plum genome. Variant detection was performed using CLC Genomics Workbench Basic Variant Detection tool with the following modifications to the default parameters: Ploidy = 6, minimum coverage = 20, minimum count = 4, minimum frequency = 8.0. Alignments of reads spanning the GAT repetitive element within exon 3 were extracted and manually refined using BioEdit (http://www.mbio.ncsu.edu/BioEdit/bioedit) to correct for errors and identify the six variant alleles.

### Tree measurements

Peach tree branch measurements were taken from 10 standard trees (*TAC/TAC1*) and 10 pillar (*tac1/tac1*) at the end of the 2012 growing season. Fifty measurements in total were used for both 2012 branch growth and diameter, with five branches measured per tree. The 2012 shoot growth (length) measurements taken from June 11 to August 14 were all from the same branches. Trunk diameters and heights were taken from 50 individual standard and pillar trees. All peach trees were from 4-year-old trees in the field that were part of a pillar/upright/standard (1:2:1) segregating population. Trunk and stem diameters were measured 4 cm from the base using calipers. Field-grown RNA interference (RNAi) and overexpression (OE) *TAC1* transgenic plums were measured in the spring of 2017 as follows: trunk and branch diameters were measured using digital calipers, with trunk diameter taken at 4 inches from the base, and branch diameters taken at the base of the branch; branch diameters, lengths, and angles were taken for the first four major branches of each tree; branch angles were measured using a protractor aligned to the main trunk and reported as degrees from vertical. Transgenic plums were compared to untransformed seedlings from their respective standard plum varieties, “Bluebyrd” or “President”. Measurements were taken for three of the RNAi lines (Lines 1, 3, and 6), all three OE lines. Between four and six replicate trees were measured per line and four branches per tree were measured for angles and lengths.

### Tree models

Tree models were acquired using a robotic vision system called Robotic System for Tree Shape Estimation, or RoTSE, which is described in detail in Tabb and Medeiros (2017a)^32^. RoTSE consists of a small truck, robot arm, cameras, generator and other incidentals. The truck was parked near the tree, and a background unit consisting of blue background material was parked on the other side of the tree. The robot arm outfitted with cameras acquired still frame images. These images were processed via algorithms described in Tabb and Medeiros (2017a), Tabb (2013), and Tabb (2014) to generate estimates of the tree’s shape, which was subsequently skeletonized with the method of Tabb and Medeiros (2017b)^32–35^.

### Plasmid construction

To generate the *TAC1*-silencing vector, PpeTAC1-HG, *PpeTAC1*-specific primer sequences Fwd 5′-TGGGTTTGCTGGGAATGTGA-3′ and Rev 5′-CAGCTGGTTTCTGAACAATGGC-3′ were used to PCR amplify a 380-base-pair cDNA fragment from peach genomic DNA. The resulting fragment was cloned into the pENTR-D TOPO (Invitrogen) vector per the manufacturer’s specifications and sequenced for verification. PpeTAC1_300 pENTR-D TOPO was recombined with the RNAi-silencing vector pHellsgate 8 (Commonwealth Scientific and Industrial Research Organisation (CSIRO), Australia) using gateway technology to create PpeTAC1-HG (Invitrogen, Carlsbad, CA). To generate the 35S::TAC1 OE vector, *PpeTAC1* primer sequences Fwd 5′-GAATTCAATTCGCTCACAAAATATGAAG-3′ and Rev 5′-CCTTGTGTGCACTGAATTAAGGATCC-3′ were used to amplify the full-length *PpeTAC1*-coding sequence from peach RNA purified from apical shoots. The resulting fragment was digested and cloned into a modified pBIN-ARS vector (called pBIN-AFRS) using the EcoRI and BamHI restriction sites behind the Cauliflower Mosaic Virus 35S promoter (vector sequence and map provided in Datafile S1).

### Plum transformation

*PpeTAC1-HG* and *35S::PpeTAC1* vectors were transformed into the *Agrobacterium tumefaciens* strain GV3101. Agrobacterium-mediated transformation of plum hypocotyl slices was performed as previously described in Petri et al. (2012). Owing to the limited availability of seeds, the RNAi-silencing construct was transformed into the cultivar “President” and the OE construct was transformed into the cultivar “Bluebyrd”. Four RNAi lines and three OE lines were obtained under Kanamycin selection, propagated via tissue culture, rooted, and transplanted to soil during 2013.

### Plum RNA extraction and real-time quantitative polymerase chain reaction (qPCR)

To confirm silencing or OE of *PpeTAC1* in transgenic plums, qPCR was performed on plum transgenic and control lines. Total RNA was extracted from apical shoots of 1-year-old, greenhouse-grown plants using the SQ Total RNA Extraction Kit (Omega Biotech, Norcross, GA) per the manufacturer’s instructions. qPCR from total RNA was performed using the SYBR Green One-Step qPCR Kit (Invitrogen, Carlsbad, CA) and run in an ABI 7900HT Sequence Detection System. Each reaction was run in triplicate using 50 ng of RNA. Transgene expression was determined from 4 to 5 independent vegetatively propagated biological replicates from each transgenic line and two control plants. Expression for the controls was the average of two biological replicates. Three technical replicates were performed for each biological replicate. Quantification was performed using a standard curve derived from a serially diluted standard RNA run in parallel. A dissociation curve was run to verify that a single desired amplified product was obtained from each reaction. The *PpeTAC1* primers used [For 5′-TTTGCCAAGAAACTCATCCCTCGC and Rev 5′-GCTGCTTCTGGCCATCTGATTTGT] were designed flanking an intron to amplify a 145 bp fragment from both the *PpeTAC1* transgene and all native *PpoTAC1* alleles.

### RNAseq experiments[experimental data is being deposited into NCBI GEO and accession number will be provided]

RNA extraction from the tips of peach tree branches (~4–6 cm in length) was performed as previously described^16,36^. Briefly, frozen tissue was ground in liquid N_2_ using a mortar and pestle and subjected to RNA extraction using the E.Z.N.A. SQ Total RNA Kit (Omega Bio-tek Inc.). RNA was prepared according to the manufacturer’s instructions with the exception that 2% PVP was added to the RCL buffer. For expression profiling studies, ~6–10 cm of each of the three shoot tips (with leaves removed) were cut from four standard and four pillar trees from the KV991636 population (described above). Approximately 4 μg of total RNA for each tree was provided to the Cornell Weill Genomics center (Ithaca, NY) for single-read 50 bp sequencing. Raw reads were trimmed to remove reads <50 bp in length. Remaining reads were aligned to the peach genome version 2 using CLC “RNAseq” function under the following settings: maximum number of hits for a read = 10, count paired reads as two = No, expression value = RPKM, no global alignment, similarity fraction = 0.95, length fraction = 0.8, mismatch cost = 2, insertion cost = 3, deletion cost = 3^37^. The “Differential Gene Expression” function was used to identify differentially expressed genes (DEGs) in a pairwise fashion. Expression means were calculated from the four biological replicates of pillar and standard, respectively. DEGs with a *P*-value ≤0.05 and having a minimum average of 5 reads in both pillar and standard genotypes were used for further analysis. The DEG list was manually categorized based on published data in order to identify coordinated changes within known biological, signaling, and/or metabolic pathways^38^.

## Results

### Pillar peach trees display pleotropic phenotypes

**Fig. 1:**
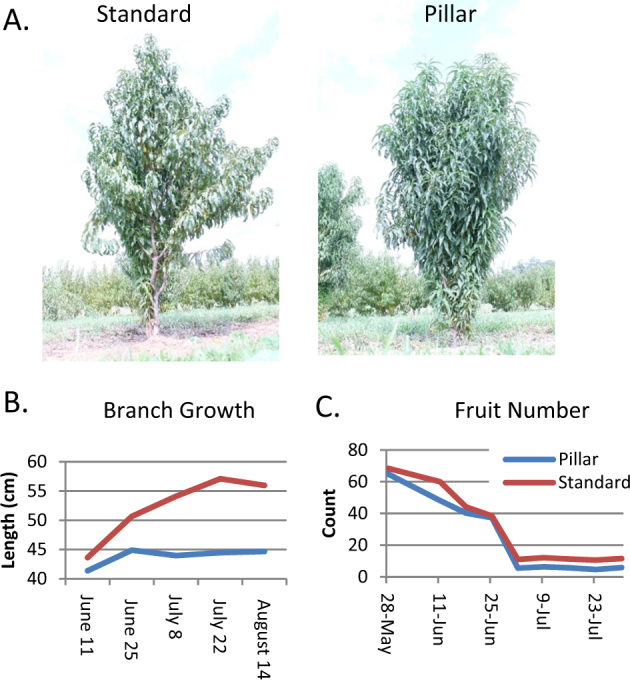
Pillar peach trees exhibit pleiotropic phenotypes. **a** Images of a standard growth habit (“Bounty”) and pillar (“Crimson Rocket”) peach trees. **b** Time course of 2013 branch growth. Note that apparent decreases in branch length throughout the season are due to small (<1 cm) differences in subsequent measurements of branches after they ceased growing. **c** Fruit set in pillar and standard trees. Note that decreases in count occur normally as a consequence of June drop

Previous studies found a number of morphological differences between pillar and standard peach growth habits in addition to branch angle including stem diameter, branch length, and internode distance^8^. In these reports, comparisons were performed between different cultivars and could not definitively account for genetic background differences that are not directly associated with the pillar trait. To directly compare between trees of the same genetic background, we measured tree height, trunk diameter, and branch length using sibling trees from a peach population segregating for the pillar trait (Figure 1 and Table 1). Pillar peaches displayed pleotropic phenotypes and had reduced trunk diameter and stem diameters and reduced branch lengths but showed no differences in overall tree height or fruit count (Table 1 and Fig. 1b, c).

**Table 1:** Growth measurements taken at the end of the 2012 growing season from 4-year-old standard and pillar trees from a segregating population in the field at the USDA Appalachian Fruit Research Station

	**Standard peach**			**Pillar peach**			***P*** **-value**
	Avg.	SD	SEM	Avg.	SD	SEM	
Branch growth in 2012 (cm)	^a^82.9	30.5	4.31	^a^65.2	35.4	5.01	0.0086
Diameter of 2012 branches (mm)	7.76	2.67	0.38	6.78	2.87	0.41	0.081
Trunk height (m)	2.97	0.323	0.05	2.97	0.303	0.04	0.99
Trunk diameter (cm)	^a^17	2.51	0.35	^a^14.5	2.46	0.35	<0.001

For each genotype, branch measurements are from 50 branches from 10 trees and trunk measurements are from 50 trees

*SD* standard deviation, *SEM* standard error of the mean

^a^Statistically a significant difference based on a *P*-value <0.05

### *TAC1*allelic variation in *Prunus domestica*

**Fig. 2:**
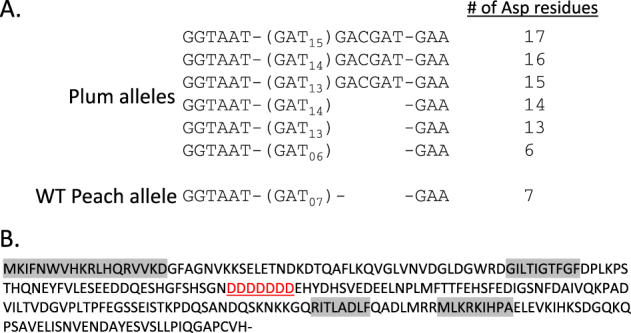
*P. domestica* carries six distinct *TAC1* alleles containing variable length GAT repeats within exon3. **a** List of allelic variants compared to the peach wild-type *TAC1* allele. **b** Amino acid sequence of *PpeTAC1*. Aspartic acid repeat is underlined. Motifs conserved in IGT proteins are highlighted in gray

To better understand the role of *TAC1* in regulating tree architecture, we evaluated *TAC1* gene function in the closely related species *P. domestica* (plum). Unlike peach, plum can be readily transformed via *Agrobacterium*-mediated strategies^39,40^. However, the use of plum is complicated by the fact that it is a hexaploid species thought to have arisen as an interspecific cross between *Prunus cerasifera* and *Prunus spinosa*^40,41^. It is also important to note that plum trees tend to have a naturally more upright architecture than peach^42,43^. To address this, we first characterized the native TAC1 alleles present in plum. The genome of the commercial plum variety “Improved French” was sequenced to an average haploid coverage of 102× via a combination of Illumina 100 bp paired-end reads and 100 bp mate-pair reads and aligned to a 5 kb fragment spanning the *TAC1* gene from peach genome version 1^31^. Next, single-nucleotide polymorphisms (SNPs) and insertion/deletion (indel) variants of the native *PdoTAC1* alleles were identified relative to the peach *TAC1* sequence. Overall, there was a high degree of sequence identity between peach and plum. A total of 16 variants were identified within coding sequences, 5 of which were tightly clustered (Table S1). Manual inspection of this region revealed that the five clustered SNPs were errantly called due to misalignment caused by the presence of a variable simple sequence repeat (SSR) located within exon 3 that comprised multiple *TAC1* alleles (Fig. 2a). Incidentally, this SSR occurred in the same location (between conserved domains II and III) as the previously identified insertion event in pillar peach (Fig. 2b)^16^. The SSR sequence consists of a repeated Asp codon (GAT_7_). Plum alleles contained repeats ranging from GAT_6_ to GAT_15_ and the longest alleles included two additional 3′ Asp codons (GAC GAT). While the impact of the additional Asp residues on TAC1 protein function is unknown, the results suggest that this region may be unstable in *Prunus* species and could contribute to phenotypic variability.

### TAC1 silenced and OX plums

Given the high level of sequence identity between the *PpeTAC1* and *PdoTAC1* sequences, we used a previously cloned peach *TAC1* cDNA sequence to create silencing (via RNAi) and OE (under the 35S promoter) constructs for transformation into plum. Four putative RNAi transgenic lines and three putative OE lines were obtained. Owing to limited seed for the hypocotyl-based transformation method, the RNAi plums were generated using the plum cultivar President, while the OE lines were generated using seed from the cultivar Bluebyrd. These two varieties differed slightly in their growth habit (Table 2). Thus the RNAi and OE lines were only evaluated with respect to their parental cultivar background.

**Table 2:** Growth measurements from TAC1 RNAi plum trees and President their respective standard plum control

	**Standard President**			**TAC1 RNAi**			***P*** **-value**
	Avg.	SD	SEM	Avg.	SD	SEM	
Branch angle	^a^38	9.5	4.8	^a^17.0	10.8	2.7	<0.01
Branch length (cm)	^a^26	22.2	11.1	^a^50.0	19.1	4.8	<0.01
Branch diameter (mm)	13	4.6	2.3	12.0	3.2	0.8	0.23
Trunk diameter (mm)	^a^34	4.5	2.3	^a^42.0	5.3	1.3	0.01
Tree height (m)	3.11	0.5	0.3	3.4	0.5	0.1	0.3

*SD* standard deviation, *SEM* standard error of the mean

^a^Statistically a significant difference based on a *P*-value <0.05

**Fig. 3:**
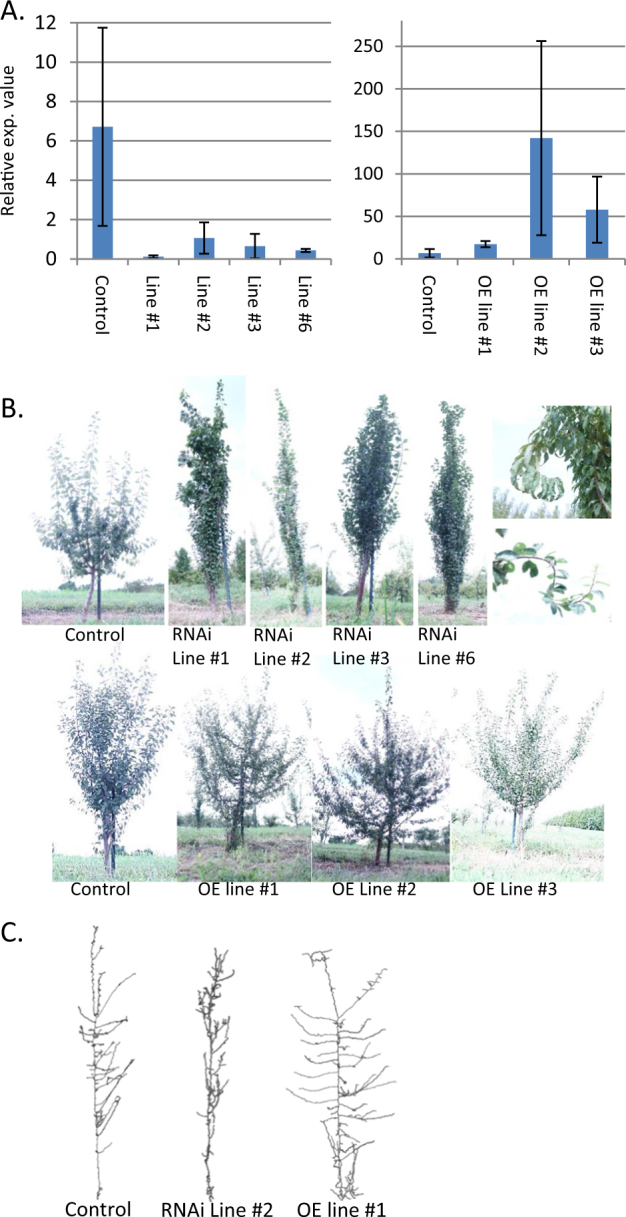
Transgenic plums with altered TAC1 expression display branch angle phenotypes. **a** qPCR results showing relative TAC1 expression in RNAi and overexpression (OE) lines. Bars represent standard deviations of biological replicates. **b** Images of 3-year field-grown RNAi (top) and OE (bottom) lines. Images on right show the occasional twisting branch phenotype observed in pillar peach (upper) and RNAi plum (lower). The control of the RNAi lines is the cultivar President and the cone for the OE lines is Bluebyrd. **c** Skeletonized representations of trees created from 3D reconstructions taken after year 2 in the field

Using primers designed to detect all *PdoTAC1* alleles, qPCR confirmed that all four RNAi lines showed significantly reduced *TAC1* expression (Fig. 3a). The 3 plum OE lines showed 2–20-fold increases in *TAC1* expression (Fig. 3a). Replicate trees rooted from tissue culture explants for each line were planted to the field under APHIS permit and monitored for phenotypes. During their second year of growth, select trees were photographed in the winter and the resulting images were used to generate three-dimensional reconstructions and skeleton images. Detailed phenotypic measurements were taken from these trees during the third growing season. All four *PpeTAC1* RNAi lines showed a clear pillar growth habit marked by vertically oriented secondary branches and significantly narrower branch angles when compared to untransformed trees of the same cultivar (Figs. 3b, c). In contrast to peach pillar trees, plum RNAi lines had significantly longer lateral branches and thicker trunks (Table 2). The RNAi lines also displayed a waving or twisting of the upper branches, a phenotype that has been occasionally observed in pillar peach (Fig. 3b**)**. When their leaves were present, the *PpeTAC1* OE lines displayed few visibly obvious architectural phenotypes. However, skeletonized images revealed that their overall architecture was different (Fig. 3c). OE lines had, on average, significantly wider branch angles, thicker stems, and were overall larger trees relative to controls (Table 3; Fig. 3b, c; Figure S1).

**Table 3:** Measurements from TAC1 OE plum trees and their respective standard plum control Bluebyrd

	**Standard Bluebyrd**			**TAC1 OE**			***P*** **-value**
	Avg.	SD	SEM	Avg.	SD	SEM	
Branch angle	^a^30	11.2	5.6	^a^44.0	13.7	3.5	<0.01
Branch length (cm)	33	19.7	9.9	45.0	28.7	7.4	0.06
Branch diameter (mm)	^a^11	3.9	2.0	^a^15.0	4.0	1.0	0.01
Trunk diameter (mm)	39	26.4	13.2	40.0	5.0	1.3	0.84
Tree height (m)	^a^2.4	0.4	0.2	^a^3.3	0.2	0.0	<0.01

*SD* standard deviation, *SEM* standard error of the mean

^a^Statistically a significant difference based on a *P*-value <0.05

### Expression profiling of pillar trees

**Fig. 4:**
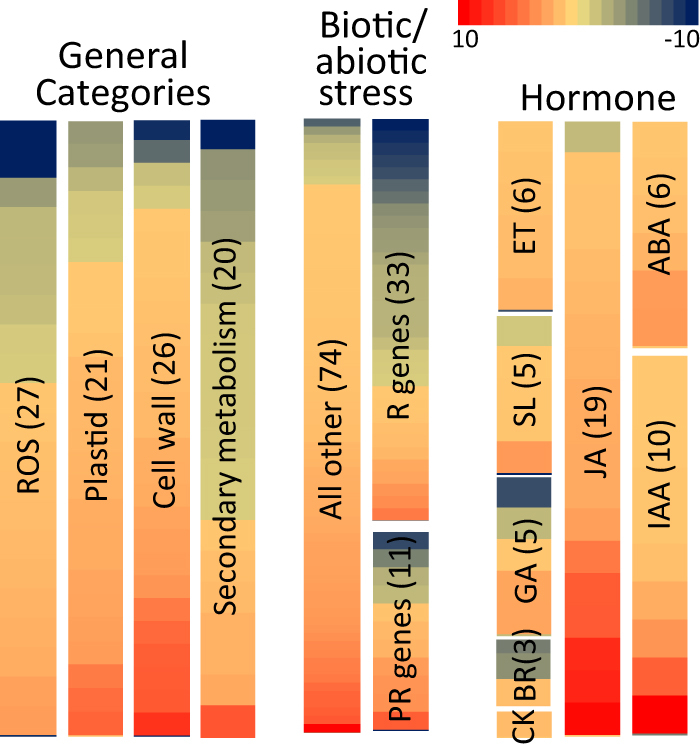
Heat maps showing DEG categories in pillar peach trees. Abbreviations used are as follows (from left to right): R genes resistance genes, PR genes pathogenesis-related genes, ROS reactive oxygen species, ET ethylene, SL strigolactone, GA giberellic acid, BR brasinolide, CK cytokinin, JA jasmonic acid, ABA abscisic acid, IAA auxin. Numbers of DEGs are shown in parentheses. Scale bar is shown in upper right (fold change)

Currently, little is known about the mechanism of action for *TAC1* in regulating branch angle. To begin to assess this, we performed an RNAseq expression profiling study using shoot tips from 4 standard and 4 pillar individuals of a segregating peach population (raw data is available for download at the NCBI Gene Expression Omnibus (GEO) accession # GSE112649). A total of 641 DEGs (*P*-value > 0.05) showing fold changes >1.5 were identified, with more genes being upregulated in pillar (453) than downregulated (188) (Dataset S1). To evaluate potential changes in regulatory and/or metabolic pathways, all DEGs were examined using MapMan^38^. Gene enrichment analysis revealed significant differential expression of genes involved in biotic and abiotic stress responses along with several other categories that are commonly associated with stress/defense pathways, including reactive oxygen, cell wall, and secondary metabolism (Fig. 4, Figure S2). Manual categorization of all DEGs revealed that nearly one third of the genes in the biotic stress category^33^ were comprised by NBS-LRR disease resistance genes (R genes) (Fig. 4). Among genes associated with hormone functions, the most were associated with jasmonic acid (JA) signaling including JA metabolism and terpene biosynthesis genes, which were universally upregulated in pillar trees. Genes associated with auxin and ethylene signaling were also largely upregulated while those related to brassinosteroid signaling were repressed (Fig. 4, Dataset S1). Other notable genes included *MORE AXILLARY GROWTH 1* (*MAX1*) and *MAX2*, which regulate branch outgrowth and were previously shown to be differentially expressed in pillar peach trees (Dataset S1)^21^. Three DEGs encoding 2OG- and Fe(II)-dependent oxygenases were identified; however, none of them appeared to be orthologous to the putative apple columnar gene *MdCo31* (Dataset S1)^11^. Also of potential functional significance was a gene we recently found to be associated with the peach weeping growth habit (*WEEP*), which was slightly downregulated in pillar trees (Hollender et al., in press).

## Discussion

Over the next half century, the Food and Agriculture Organization of the United Nations predicts that meeting the growing demand for food will require substantial increases to crop productivity without concomitant expansion of total farm acreage^44^. Crops with reduced levels of *TAC1* expression could help meet this challenge having demonstrated potential for narrowing plant architectural profiles and increasing productivity via high-density planting. Here we evaluated the effect of altered *TAC1* expression in both peach and plum trees.

Segregating pillar peach siblings carrying a knockout mutation of *PpeTAC1* had consistently shorter branches and thinner trunks and lateral branches while tree height was unchanged. Fruit yield showed no significant differences between pillar and standard trees. These data confirm a previous report that the pillar cultivar “Crimson Rocket” produced shorter and fewer sylleptic branches compared to a standard peach cultivar and that differences in branch growth rate occurred primarily in spring^8^.

We previously discovered that “Italian” pillar peach trees have a repetitive element inserted within a repetitive region of *PpeTAC1* Exon 3^16^. Surprisingly, Illumina DNA sequencing of the plum (*P. domestica*) cultivar “Improved French” revealed the presence of a variable length GAT repeat in this same location resulting in six distinct alleles within the hexaploid genome. These changes resulted in some alleles encoding long runs of aspartic acid residues 13–17 amino acids in length. A single shorter allele (GAT_6_) was similar in length to that found in peach (GAT_7_). Coincidentally, many plum cultivars including “Improved French”, “President”, and “Bluebyrd” plum (controls depicted in Fig. 3b), have a more upright stature than commonly observed in other *Prunus* species^42,43^. We speculate that the additional Asp residues in *P. domestica* alleles may result in TAC1 proteins that have reduced function or are less stable leading to a more upright tree shape. In animals, these genetic “stutters” within coding sequences have been shown to underlie genetic disorders such as Huntington’s disease where CAG repeat expansion produces longer tracts of glutamines within the huntingtin protein leading to protein instability^45^.

Silencing of *PdoTAC1* via RNAi resulted in plum trees having architectural profiles that were significantly narrower than those observed in peach. Branch growth in these trees was extremely vertical producing slender but dense canopies. Mean branch angles across all RNAi plum lines were 17^o^ versus pillar peaches, which have been reported to be as wide as 40^o^^46^. The RNAi plum lines also exhibited additional differences from the pillar peach including longer branch lengths and thicker trunk diameters. In contrast, transgenic plums overexpressing *PpeTAC1* displayed wider branch angles, thicker branches, and increased overall tree height relative to non-transgenic controls. Given the lack of knowledge about the cellular function of *TAC1*, it is difficult to conceive of a model by which these diverse phenotypic effects could be explained aside from generalities regarding potential changes in levels of hormones or their translocation, perception, and/or signaling. A prior study attributed some of the phenotypic variation within pillar peaches to higher auxin levels and higher auxin–cytokinin ratios found throughout the canopy^8^. The contrasting results in plum suggest that TAC1 RNAi plums may have hormone profiles that are either different from peach or that the phenotypic effects result from other unknown genetic or physiological variables. Collectively, these data show that biotechnology strategies can be used to engineer tree shapes by altering *TAC1* levels; however, these may be accompanied by additional phenotypic effects that are less predictable.

Expression profiling data support a hypothesis that loss of *TAC1* leads to changes in key plant hormones as responses to auxin and jasmonic acid were consistently upregulated. Surprisingly, pillar trees exhibited pronounced changes in biotic/abiotic stress signaling including numerous R genes and pathogenesis-related genes. This effect may be caused by or associated with the upregulation of JA metabolism and/or signaling, which is a known defense hormone. Categories related to defense/stress responses were also differentially expressed including reactive oxygen, secondary metabolism, and cell wall biosynthesis. Overall, these expression changes are similar to what was previously reported for apple columnar trees, which exhibited changes in genes associated with cell wall metabolism, defense, and JA responses^47,48^. At this time, we cannot rule out the possibility that the *TAC1* locus is tightly linked to genetic variation within an unrelated gene that affects biotic stress signaling pathways. While the data support the possibility that *TAC1* functions via altered defense hormone profiles and/or translocation, additional in-depth molecular studies will be needed to better understand these relationships.

## Electronic supplementary material

Table S1

Figure S1, Figure S2

Datafile S1

Dataset S1

## References

[1] Vidovič J (1974). Effect of the change of leaf angle arrangement on productivity of maize (*Zea mays* L.) stands. Biol. Plant.

[2] Tan L (2008). Control of a key transition from prostrate to erect growth in rice domestication. Nat. Genet..

[3] Miller S, Scorza R (2002). Training and performance of pillar, upright, and standard form peach trees-early results. Acta Hortic..

[4] Glenn DM, Tworkoski T, Scorza R, Miller SS (2011). Long-term effects of peach production systems for standard and pillar growth types on yield and economic parameters. Hort. Technol..

[5] Scorza R, Bassi D, Rizzo M (2000). Developing new peach tree growth habits for higher density plantings. In 42nd Annual IDFTA Conference.

[6] Giovannini D, Glenn DM, Scorza R, Welker WV (1994). Dry matter distribution of three peach growth types. HortScience.

[7] Glenn DM, Bassett CB, Tworkoski T, Scorza R, Miller SS (2015). Tree architecture of pillar and standard peach affect canopy transpiration and water use efficiency. Sci. Hortic. (Amst.)..

[8] Tworkoski T, Miller S, Scorza R (2006). Relationship of pruning and growth morphology with hormone ratios in shoots of Pillar and Standard peach trees. J. Plant Growth Regul..

[9] Tobutt KR (1994). Combining apetalous parthenocarpy with columnar growth habit in apple. Euphytica [Internet]..

[10] Petersen R, Krost C (2013). Tracing a key player in the regulation of plant architecture: The columnar growth habit of apple trees (*Malus domestica*). Planta.

[11] Wolters PJ, Schouten HJ, Velasco R, Si-Ammour A, Baldi P (2013). Evidence for regulation of columnar habit in apple by a putative 2OG-Fe(II) oxygenase. New Phytol..

[12] Okada K (2016). Expression of a putative dioxygenase gene adjacent to an insertion mutation is involved in the short internodes of columnar apples (*Malus domestica*). J. Plant Res..

[13] Yu B (2007). TAC1, a major quantitative trait locus controlling tiller angle in rice. Plant J..

[14] Wu X, Tang D, Li M, Wang K, Cheng Z (2013). Loose Plant Architecture1, an INDETERMINATE DOMAIN protein involved in shoot gravitropism, regulates plant architecture in rice. Plant Physiol..

[15] Tanimoto M, Tremblay R, Colasanti J (2008). Altered gravitropic response, amyloplast sedimentation and circumnutation in the Arabidopsis shoot gravitropism 5 mutant are associated with reduced starch levels. Plant Mol. Biol..

[16] Dardick C (2013). PpeTAC1 promotes the horizontal growth of branches in peach trees and is a member of a functionally conserved gene family found in diverse plants species. Plant J..

[17] Li H (2017). Genome-wide association mapping reveals the genetic control underlying branch angle in rapeseed (*Brassica napus* L.). Front. Plant Sci..

[18] Zhao H (2014). Natural variation and genetic analysis of the tiller angle gene MsTAC1 in *Miscanthus sinensis*. Planta.

[19] Xu D (2017). PzTAC and PzLAZY from a narrow-crown poplar contribute to regulation of branch angles. Plant Physiol. Biochem..

[20] Ku L (2011). Cloning and characterization of a putative tac1 ortholog associated with leaf angle in maize (zea mays l. PLoS ONE.

[21] Tworkoski T, Webb K, Callahan A (2015). Auxin levels and MAX1–4 and TAC1 gene expression in different growth habits of peach. Plant Growth Regul..

[22] Hollender CA, Dardick C (2015). Molecular basis of angiosperm tree architecture. New Phytol..

[23] Guseman JM, Webb K, Srinivasan C, Dardick C (2017). DRO1 influences root system architecture in Arabidopsis and Prunus species. Plant J..

[24] Yoshihara T, Iino M (2007). Identification of the gravitropism-related rice gene LAZY1 and elucidation of LAZY1-dependent and -independent gravity signaling pathways. Plant Cell Physiol..

[25] Li P (2007). LAZY1 controls rice shoot gravitropism through regulating polar auxin transport. Cell Res..

[26] Yoshihara T, Spalding EP, Iino M (2013). AtLAZY1 is a signaling component required for gravitropism of the *Arabidopsis thaliana* inflorescence. Plant J..

[27] Uga Y (2013). Control of root system architecture by DEEPER ROOTING 1 increases rice yield under drought conditions. Nat. Genet..

[28] Taniguchi M (2017). The Arabidopsis LAZY1 family plays a key role in gravity signaling within statocytes and in branch angle control of roots and shoots. Plant Cell.

[29] Yoshihara T, Spalding EP (2017). LAZY genes mediate the effects of gravity on auxin gradients and plant architecture. Plant Physiol..

[30] Dong Z (2013). Maize LAZY1 mediates shoot gravitropism and inflorescence development through regulating auxin transport, auxin signaling, and light response. Plant Physiol..

[31] Verde I (2013). The high-quality draft genome of peach (*Prunus persica*) identifies unique patterns of genetic diversity, domestication and genome evolution. Nat. Genet..

[32] Tabb, A. & Medeiros, H. A robotic vision system to measure tree traits. In *Proc. IEEE/RSJ International Conference on Intelligent Robots and Systems* 6005–6012 (2017).

[33] Tabb, A. Shape from silhouette probability maps: reconstruction of thin objects in the presence of silhouette extraction and calibration error. In *Proc IEEE Computer Society Conference on Computer Vision and Pattern Recognition* IEEE, Portland, OR, USA. 161–168 (2013) 10.1109/CVPR.2013.28.

[34] Tabb, A. *Shape from Inconsistent Silhouette: Reconstructions of Objects in the Presence of Segmentation and Calibration Error*. (Purdue University; West Lafayette, Indiana, USA. 2014).

[35] Tabb, A. & Medeiros, H. Fast and robust curve skeletonization for real-world elongated objects. In *Proc. IEEE Winter Conference on Applications of Computer Vision* 1935–1943 (2018).

[36] Hollender CA, Hadiarto T, Srinivasan C, Scorza R, Dardick C (2016). A brachytic dwarfism trait (dw) in peach trees is caused by a nonsense mutation within the gibberellic acid receptor PpeGID1c. New Phytol..

[37] Verde I (2017). The Peachv2.0 release: high-resolution linkage mapping and deep resequencing improve chromosome-scale assembly and contiguity. BMC Genomics.

[38] Thimm O (2004). mapman: a user driven tool to display genomics data sets onto diagrams of metabolic pathways and other biological processes. Plant J..

[39] Petri C, Webb K, Dardick C, Scorza R (2009). A high-throughput transformation system in plum (*Prunus domestica* L.) useful for functional genomics in Rosaceae. Acta Hortic..

[40] Petri C, Webb K, Hily JM, Dardick C, Scorza R (2008). High transformation efficiency in plum (*Prunus domestica* L.): a new tool for functional genomics studies in *Prunus* spp. Mol. Breed..

[41] Bortiri E (2001). Phylogeny and systematics of *Prunus* (Rosaceae) as determined by sequence analysis of ITS and the chloroplast *trnL-trnF* spacer DNA. Syst. Bot..

[42] Faust, M. & Surányi, D. in *Horticultural Reviews* (eds Jules Janick) Ch. 4 (Wiley, Oxford, 2010).

[43] Rieger, M. in *Introduction to Fruit Crops* (eds Amarjit S. Basra, PhD) Ch. 28 (Food Products Press, New York, 2006).

[44] Alexandratos, N. & Bruinsma, J. *World Agriculture Towards 2030/2050: The 2012 Revision* (ESA Working paper No. 12-03. Rome, FAO. 2012).

[45] Nørremølle A (1993). Trinucleotide repeat elongation in the Huntingtin gene in Huntington disease patients from 71 Danish families. Hum. Mol. Genet..

[46] Thomas T, Ralph S (2001). Root and shoot characteristics of peach trees with different growth habits. J. Am. Soc. Hortic. Sci..

[47] Krost C, Petersen R, Schmidt ER (2012). The transcriptomes of columnar and standard type apple trees (Malus x domestica) — a comparative study. Gene.

[48] Petersen R, Djozgic H, Rieger B, Rapp S, Schmidt E (2015). Columnar apple primary roots share some features of the columnar-specific gene expression profile of aerial plant parts as evidenced by RNA-Seq analysis. BMC Plant Biol..

