# Supplementary material for: Alteration of TAC1 expression in Prunus species leads to pleiotropic shoot phenotypes
Source: Hortic Res. 2018 May 1;5:26. doi: 10.1038/s41438-018-0034-1 (PMC5928093; doi:10.1038/s41438-018-0034-1)
Supplement: Supplementary file 2 — Figure S1, Figure S2 [file 41438_2018_34_MOESM2_ESM.pptx]

## Slide 1
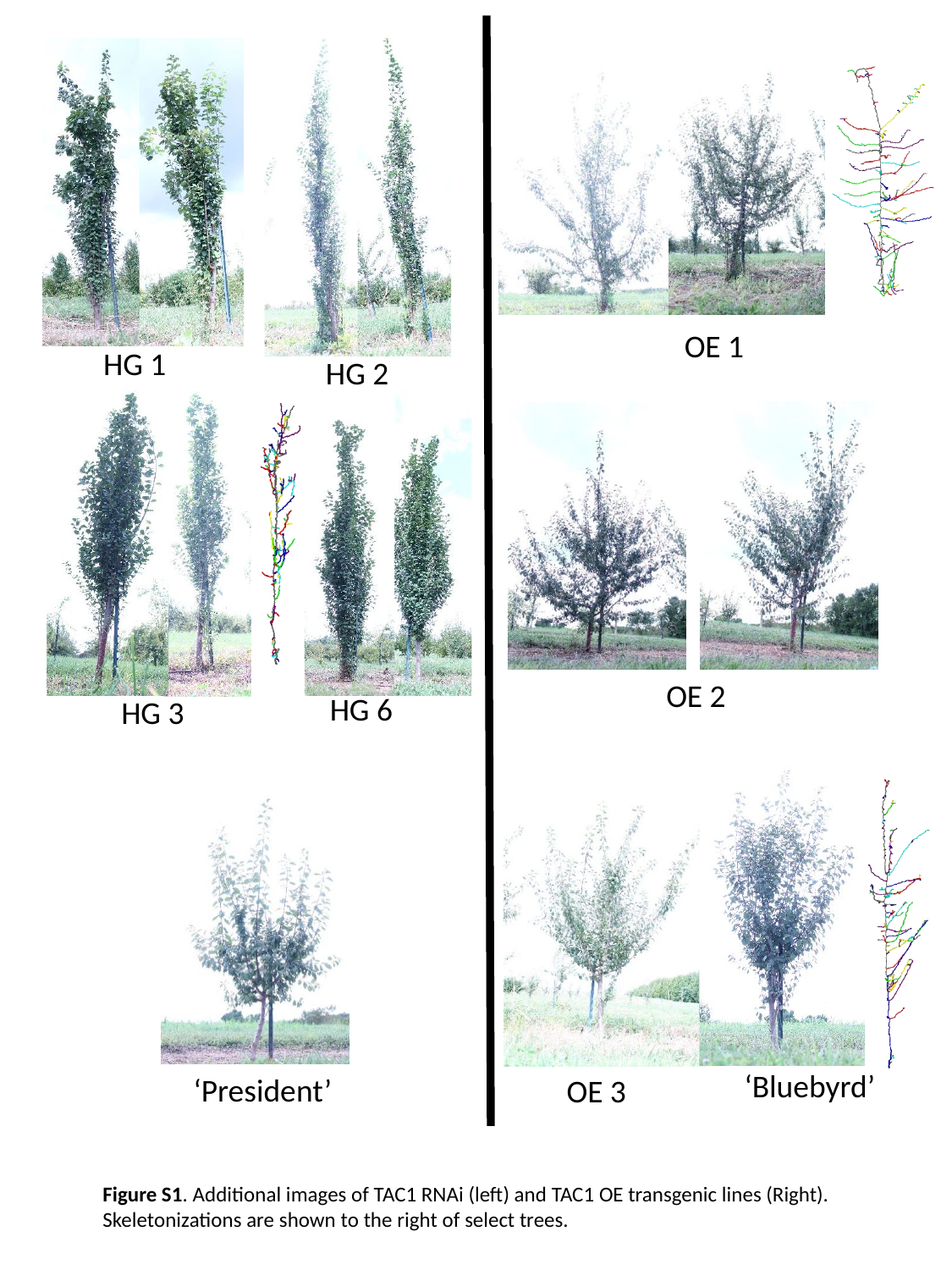

OE 1
HG 1
HG 2
OE 2
HG 6
HG 3
‘Bluebyrd’
‘President’
OE 3
Figure S1. Additional images of TAC1 RNAi (left) and TAC1 OE transgenic lines (Right). Skeletonizations are shown to the right of select trees.

## Slide 2
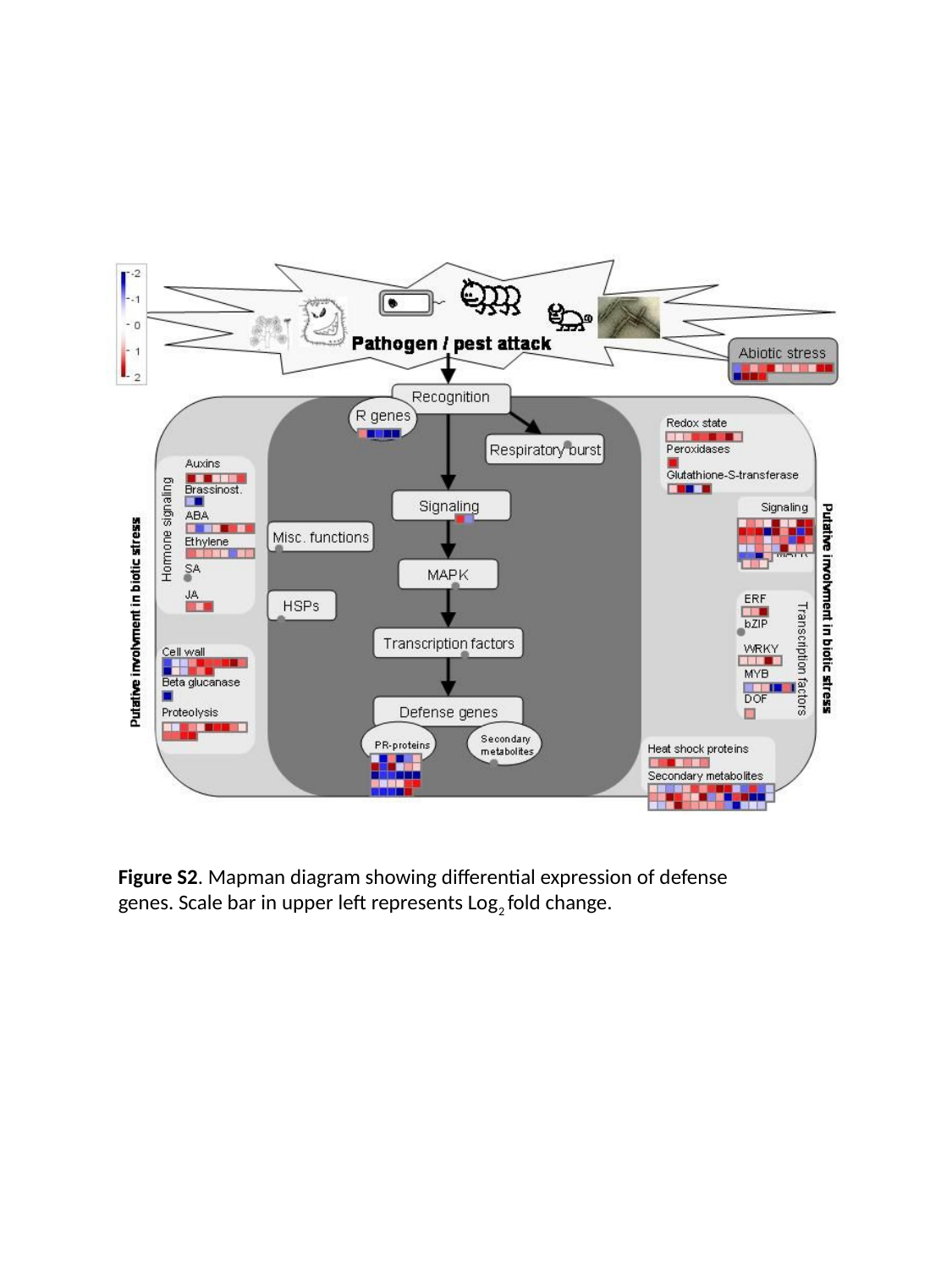

Figure S2. Mapman diagram showing differential expression of defense genes. Scale bar in upper left represents Log2 fold change.
