# Supplementary material for: Alteration of TAC1 expression in Prunus species leads to pleiotropic shoot phenotypes
Source: Hortic Res. 2018 May 1;5:26. doi: 10.1038/s41438-018-0034-1 (PMC5928093; doi:10.1038/s41438-018-0034-1)

**pBIN AFRS +35S Complete Vector Sequence:**

CCGGGCTGGTTGCCCTCGCCGCTGGGCTGGCGGCCGTCTATGGCCCTGCAAACGCGCCAGAAACGCCGTCGAAGCCGTGTGCGAGACACCGCGGCCGCCGGCGTTGTGGATACCTCGCGGAAAACTTGGCCCTCACTGACAGATGAGGGGCGGACGTTGACACTTGAGGGGCCGACTCACCCGGCGCGGCGTTGACAGATGAGGGGCAGGCTCGATTTCGGCCGGCGACGTGGAGCTGGCCAGCCTCGCAAATCGGCGAAAACGCCTGATTTTACGCGAGTTTCCCACAGATGATGTGGACAAGCCTGGGGATAAGTGCCCTGCGGTATTGACACTTGAGGGGCGCGACTACTGACAGATGAGGGGCGCGATCCTTGACACTTGAGGGGCAGAGTGCTGACAGATGAGGGGCGCACCTATTGACATTTGAGGGGCTGTCCACAGGCAGAAAATCCAGCATTTGCAAGGGTTTCCGCCCGTTTTTCGGCCACCGCTAACCTGTCTTTTAACCTGCTTTTAAACCAATATTTATAAACCTTGTTTTTAACCAGGGCTGCGCCCTGTGCGCGTGACCGCGCACGCCGAAGGGGGGTGCCCCCCCTTCTCGAACCCTCCCGGCCCGCTAACGCGGGCCTCCCATCCCCCCAGGGGCTGCGCCCCTCGGCCGCGAACGGCCTCACCCCAAAAATGGCAGCGCTGGCAGTCCTTGCCATTGCCGGGATCGGGGCAGTAACGGGATGGGCGATCAGCCCGAGCGCGACGCCCGGAAGCATTGACGTGCCGCAGGTGCTGGCATCGACATTCAGCGACCAGGTGCCGGGCAGTGAGGGCGGCGGCCTGGGTGGCGGCCTGCCCTTCACTTCGGCCGTCGGGGCATTCACGGACTTCATGGCGGGGCCGGCAATTTTTACCTTGGGCATTCTTGGCATAGTGGTCGCGGGTGCCGTGCTCGTGTTCGGGGGTGCGATAAACCCAGCGAACCATTTGAGGTGATAGGTAAGATTATACCGAGGTATGAAAACGAGAATTGGACCTTTACAGAATTACTCTATGAAGCGCCATATTTAAAAAGCTACCAAGACGAAGAGGATGAAGAGGATGAGGAGGCAGATTGCCTTGAATATATTGACAATACTGATAAGATAATATATCTTTTATATAGAAGATATCGCCGTATGTAAGGATTTCAGGGGGCAAGGCATAGGCAGCGCGCTTATCAATATATCTATAGAATGGGCAAAGCATAAAAACTTGCATGGACTAATGCTTGAAACCCAGGACAATAACCTTATAGCTTGTAAATTCTATCATAATTGGGTAATGACTCCAACTTATTGATAGTGTTTTATGTTCAGATAATGCCCGATGACTTTGTCATGCAGCTCCACCGATTTTGAGAACGACAGCGACTTCCGTCCCAGCCGTGCCAGGTGCTGCCTCAGATTCAGGTTATGCCGCTCAATTCGCTGCGTATATCGCTTGCTGATTACGTGCAGCTTTCCCTTCAGGCGGGATTCATACAGCGGCCAGCCATCCGTCATCCATATCACCACGTCAAAGGGTGACAGCAGGCTCATAAGACGCCCCAGCGTCGCCATAGTGCGTTCACCGAATACGTGCGCAACAACCGTCTTCCGGAGACTGTCATACGCGTAAAACAGCCAGCGCTGGCGCGATTTAGCCCCGACATAGCCCCACTGTTCGTCCATTTCCGCGCAGACGATGACGTCACTGCCCGGCTGTATGCGCGAGGTTACATATGCGGTGTGAAATACCGCACAGATGCGTAAGGAGAAAATACCGCATCAGGCGCTCTTCCGCTTCCTCGCTCACTGACTCGCTGCGCTCGGTCGTTCGGCTGCGGCGAGCGGTATCAGCTCACTCAAAGGCGGTAATACGGTTATCCACAGAATCAGGGGATAACGCAGGAAAGAACATGTGAGCAAAAGGCCAGCAAAAGGCCAGGAACCGTAAAAAGGCCGCGTTGCTGGCGTTTTTCCATAGGCTCCGCCCCCCTGACGAGCATCACAAAAATCGACGCTCAAGTCAGAGGTGGCGAAACCCGACAGGACTATAAAGATACCAGGCGTTTCCCCCTGGAAGCTCCCTCGTGCGCTCTCCTGTTCCGACCCTGCCGCTTACCGGATACCTGTCCGCCTTTCTCCCTTCGGGAAGCGTGGCGCTTTCTCATAGCTCACGCTGTAGGTATCTCAGTTCGGTGTAGGTCGTTCGCTCCAAGCTGGGCTGTGTGCACGAACCCCCCGTTCAGCCCGACCGCTGCGCCTTATCCGGTAACTATCGTCTTGAGTCCAACCCGGTAAGACACGACTTATCGCCACTGGCAGCAGCCAGTTACCGACTGCGGCCTGAGTTTTTTAAGTGACGTAAAATCGTGTTGAGGCCAACGCCCATAATGCGGGCTGTTGCCCGGCATCCAACGCCATTCATGGCCATATCAATGATTTTCTGGTGCGTACCGGGTTGAGAAGCGGTGTAAGTGAACTGCAGTTGCCATGTTTTACGGCAGTGAGAGCAGAGATAGCGCTGATGTCCGGCGGTGCTTTTGCCGTTACGCACCACCCCGTCAGTAGCTGAACAGGAGGGACAGCTGATAGACACAGAAGCCACTGGAGCACCTCAAAAACACCATCATACACTAAATCAGTAAGTTGGCAGCATCACCCATAATTGTGGTTTCAAAATCGGCTCCGTCGATACTATGTTATACGCCAACTTTGAAAACAACTTTGAAAAAGCTGTTTTCTGGTATTTAAGGTTTTAGAATGCAAGGAACAGTGAATTGGAGTTCGTCTTGTTATAATTAGCTTCTTGGGGTATCTTTAAATACTGTAGAAAAGAGGAAGGAAATAATAAATGGCTAAAATGAGAATATCACCGGAATTGAAAAAACTGATCGAAAAATACCGCTGCGTAAAAGATACGGAAGGAATGTCTCCTGCTAAGGTATATAAGCTGGTGGGAGAAAATGAAAACCTATATTTAAAAATGACGGACAGCCGGTATAAAGGGACCACCTATGATGTGGAACGGGAAAAGGACATGATGCTATGGCTGGAAGGAAAGCTGCCTGTTCCAAAGGTCCTGCACTTTGAACGGCATGATGGCTGGAGCAATCTGCTCATGAGTGAGGCCGATGGCGTCCTTTGCTCGGAAGAGTATGAAGATGAACAAAGCCCTGAAAAGATTATCGAGCTGTATGCGGAGTGCATCAGGCTCTTTCACTCCATCGACATATCGGATTGTCCCTATACGAATAGCTTAGACAGCCGCTTAGCCGAATTGGATTACTTACTGAATAACGATCTGGCCGATGTGGATTGCGAAAACTGGGAAGAAGACACTCCATTTAAAGATCCGCGCGAGCTGTATGATTTTTTAAAGACGGAAAAGCCCGAAGAGGAACTTGTCTTTTCCCACGGCGACCTGGGAGACAGCAACATCTTTGTGAAAGATGGCAAAGTAAGTGGCTTTATTGATCTTGGGAGAAGCGGCAGGGCGGACAAGTGGTATGACATTGCCTTCTGCGTCCGGTCGATCAGGGAGGATATCGGGGAAGAACAGTATGTCGAGCTATTTTTTGACTTACTGGGGATCAAGCCTGATTGGGAGAAAATAAAATATTATATTTTACTGGATGAATTGTTTTAGTACCTAGATGTGGCGCAACGATGCCGGCGACAAGCAGGAGCGCACCGACTTCTTCCGCATCAAGTGTTTTGGCTCTCAGGCCGAGGCCCACGGCAAGTATTTGGGCAAGGGGTCGCTGGTATTCGTGCAGGGCAAGATTCGGAATACCAAGTACGAGAAGGACGGCCAGACGGTCTACGGGACCGACTTCATTGCCGATAAGGTGGATTATCTGGACACCAAGGCACCAGGCGGGTCAAATCAGGAATAAGGGCACATTGCCCCGGCGTGAGTCGGGGCAATCCCGCAAGGAGGGTGAATGAATCGGACGTTTGACCGGAAGGCATACAGGCAAGAACTGATCGACGCGGGGTTTTCCGCCGAGGATGCCGAAACCATCGCAAGCCGCACCGTCATGCGTGCGCCCCGCGAAACCTTCCAGTCCGTCGGCTCGATGGTCCAGCAAGCTACGGCCAAGATCGAGCGCGACAGCGTGCAACTGGCTCCCCCTGCCCTGCCCGCGCCATCGGCCGCCGTGGAGCGTTCGCGTCGTCTCGAACAGGAGGCGGCAGGTTTGGCGAAGTCGATGACCATCGACACGCGAGGAACTATGACGACCAAGAAGCGAAAAACCGCCGGCGAGGACCTGGCAAAACAGGTCAGCGAGGCCAAGCAGGCCGCGTTGCTGAAACACACGAAGCAGCAGATCAAGGAAATGCAGCTTTCCTTGTTCGATATTGCGCCGTGGCCGGACACGATGCGAGCGATGCCAAACGACACGGCCCGCTCTGCCCTGTTCACCACGCGCAACAAGAAAATCCCGCGCGAGGCGCTGCAAAACAAGGTCATTTTCCACGTCAACAAGGACGTGAAGATCACCTACACCGGCGTCGAGCTGCGGGCCGACGATGACGAACTGGTGTGGCAGCAGGTGTTGGAGTACGCGAAGCGCACCCCTATCGGCGAGCCGATCACCTTCACGTTCTACGAGCTTTGCCAGGACCTGGGCTGGTCGATCAATGGCCGGTATTACACGAAGGCCGAGGAATGCCTGTCGCGCCTACAGGCGACGGCGATGGGCTTCACGTCCGACCGCGTTGGGCACCTGGAATCGGTGTCGCTGCTGCACCGCTTCCGCGTCCTGGACCGTGGCAAGAAAACGTCCCGTTGCCAGGTCCTGATCGACGAGGAAATCGTCGTGCTGTTTGCTGGCGACCACTACACGAAATTCATATGGGAGAAGTACCGCAAGCTGTCGCCGACGGCCCGACGGATGTTCGACTATTTCAGCTCGCACCGGGAGCCGTACCCGCTCAAGCTGGAAACCTTCCGCCTCATGTGCGGATCGGATTCCACCCGCGTGAAGAAGTGGCGCGAGCAGGTCGGCGAAGCCTGCGAAGAGTTGCGAGGCAGCGGCCTGGTGGAACACGCCTGGGTCAATGATGACCTGGTGCATTGCAAACGCTAGGGCCTTGTGGGGTCAGTTCCGGCTGGGGGTTCAGCAGCCAGCGCTTTACTGGCATTTCAGGAACAAGCGGGCACTGCTCGACGCACTTGCTTCGCTCAGTATCGCTCGGGACGCACGGCGCGCTCTACGAACTGCCGATAAACAGAGGATTAAAATTGACAATTGTGATTAAGGCTCAGATTCGACGGCTTGGAGCGGCCGACGTGCAGGATTTCCGCGAGATCCGATTGTCGGCCCTGAAGAAAGCTCCAGAGATGTTCGGGTCCGTTTACGAGCACGAGGAGAAAAAGCCCATGGAGGCGTTCGCTGAACGGTTGCGAGATGCCGTGGCATTCGGCGCCTACATCGACGGCGAGATCATTGGGCTGTCGGTCTTCAAACAGGAGGACGGCCCCAAGGACGCTCACAAGGCGCATCTGTCCGGCGTTTTCGTGGAGCCCGAACAGCGAGGCCGAGGGGTCGCCGGTATGCTGCTGCGGGCGTTGCCGGCGGGTTTATTGCTCGTGATGATCGTCCGACAGATTCCAACGGGAATCTGGTGGATGCGCATCTTCATCCTCGGCGCACTTAATATTTCGCTATTCTGGAGCTTGTTGTTTATTTCGGTCTACCGCCTGCCGGGCGGGGTCGCGGCGACGGTAGGCGCTGTGCAGCCGCTGATGGTCGTGTTCATCTCTGCCGCTCTGCTAGGTAGCCCGATACGATTGATGGCGGTCCTGGGGGCTATTTGCGGAACTGCGGGCGTGGCGCTGTTGGTGTTGACACCAAACGCAGCGCTAGATCCTGTCGGCGTCGCAGCGGGCCTGGCGGGGGCGGTTTCCATGGCGTTCGGAACCGTGCTGACCCGCAAGTGGCAACCTCCCGTGCCTCTGCTCACCTTTACCGCCTGGCAACTGGCGGCCGGAGGACTTCTGCTCGTTCCAGTAGCTTTAGTGTTTGATCCGCCAATCCCGATGCCTACAGGAACCAATGTTCTCGGCCTGGCGTGGCTCGGCCTGATCGGAGCGGGTTTAACCTACTTCCTTTGGTTCCGGGGGATCTCGCGACTCGAACCTACAGTTGTTTCCTTACTGGGCTTTCTCAGCCCCAGATCT

GGGGAACCCTGTGGTTGGCATGCACATACAAATGGACGAACGGATAAACCTTTTCACGCCCTTTTAAATATCCGATTATTCTAATAAACGCTCTTTTCTCTTAG GGCCGGCC

ACCCTGATAAATGCTTCAATAATATTGAAAAAGGAAGAGTATGAGTATTCAACATTTCCGTGTCGCCCTTATTCCCTTTTTTGCGGCATTTTGCCTTCCTGTTTTTGCTCACCCAGAAACGCTGGTGAAAGTAAAAGATGCTGAAGATCAGTTGGGTGCACGAGTGGGTTACATCGAACTGGATCTCAACAGCGGTAAGATCCTTGAGAGTTTTCGCCCCGAAGAACGTTTTCCAATGATGAGCACTTTTAAAGTTCTGCTATGTGGCGCGGTATTATCCCGTATTGACGCCGGGCAAGAGCAACTCGGTCGCCGCATACACTATTCTCAGAATGACTTGGTTGAGTACTCACCAGTCACAGAAAAGCATCTTACGGATGGCATGACAGTAAGAGAATTATGCAGTGCTGCCATAACCATGAGTGATAACACTGCGGCCAACTTACTTCTGACAACGATCGGAGGACCGAAGGAGCTAACCGCTTTTTTGCACAACATGGGGGATCATGTAACTCGCCTTGATCGTTGGGAACCGGAGCTGAATGAAGCCATACCAAACGACGAGCGTGACACCACGATGCCTGTAGCAATGGCAACAACGTTGCGCAAACTATTAACTGGCGAACTACTTACTCTAGCTTCCCGGCAACAATTAATAGACTGGATGGAGGCGGATAAAGTTGCAGGACCACTTCTGCGCTCGGCCCTTCCGGCTGGCTGGTTTATTGCTGATAAATCTGGAGCCGGTGAGCGTGGGTCTCGCGGTATCATTGCAGCACTGGGGCCAGATGGTAAGCCCTCCCGTATCGTAGTTATCTACACGACGGGGAGTCAGGCAACTATGGATGAACGAAATAGACAGATCGCTGAGATAGGTGCCTCACTGATTAAGCATTGGTAA

AAAACGCGTATGCCGGCGTTTACCCGCCAATATATCCTGTCA

GCGGCCGCTAACTAGTTAGGCGCGCC ACTAGAGCCAAGCTGATCTCCTTTGCCCCGGAGATCACCATGGACGACTTTCTCTATCTCTACGATCTAGGAAGAAAGTTCGACGGAGAAGGTGACGATACCATGTTCACCACCGATAATGAGAAGATTAGCCTCTTCAATTTCAGAAAGAATGCTGACCCACAGATGGTTAGAGAGGCCTACGCGGCAGGTCTCATCAAGACGATCTACCCGAGTAATAATCTCCAGGAGATCAAATACCTTCCCAAGAAGGTTAAAGATGCAGTCAAAAGATTCAGGACTAACTGCATCAAGAACACAGAGAAAGATATATTTCTCAAGATCAGAAGTACTATTCCAGTATGGACGATTCAAGGCTTGCTTCATAAACCAAGGCAAGTAATAGAGATTGGAGTCTCTAAGAAAGTAGTTCCTACTGAATCAAAGGCCATGGAGTCAAAAATTCAGATCGAGGATCTAACAGAACTCGCCGTGAAGACTGGCGAACAGTTCATACAGAGTCTTTTACGACTCAATGACAAGAAGAAAATCTTCGTCAACATGGTGGAGCACGACACTCTCGTCTACTCCAAGAATATCAAAGATACAGTCTCAGAAGACCAAAGGGCTATTGAGACTTTTCAACAAAGGGTAATATCGGGAAACCTCCTCGGATTCCATTGCCCAGCTATCTGTCACTTCATCAAAAGGACAGTAGAAAAGGAAGGTGGCACCTACAAATGCCATCATTGCGATAAAGGAAAGGCTATCGTTCAAGATGCCTCTGCCGACAGTGGTCCCAAAGATGGACCCCCACCCACGAGGAGCATCGTGGAAAAAGAAGACGTTCCAACCACGTCTTCAAAGCAAGTGGATTGATGTGATATCTCCACTGACGTAAGGGATGACGCACAATCCCACTATCCTTCGCAAGACCCTTCCTCTATATAAGGAAGTTCATTTCATTTGGAGAGGACTCCGGTATTTTTACAACAATACCACAACAAAACAAACAACAAACAACATTACAATTTACTATTCTAGTCGA GTCGACATAGGCCTATTGTACATAGTTAACTAGACGTCATTCCGGATAGATATCATAAGCTTTA**GAATTC**ATGAGCTCAT

TTAACTATAACGGTCCTAAGGT

**AGGTAAAACCAATCACACACTACTTTTAAATTTCTCTGAACATAATTAATTTGAACATAATGCTAGCTAGCTAGCTGGTGCTTGTTCTTTAGAGAGATTAATTTACATGAAATATGCAGGA**

TAGGGATAACAGGGTAAT

**GGATCC**ATTCTAGATAGCGATCGCATACCGGTATCCCGGGACCTGCAGGTTCAATTGTA

GTTTAAACTGATTTTAATGTTTAGCAAATGTCTTATCAGTTTTCTCTTTTTGTCGAACGGTAATTTAGAGTTTTTTTTGCTATATGGATTTTCGTTTTTGATGTATGTGACAACCCTCGGGATTGTTGATTTATTTCAAAACTAAGAGTTTTTGTCTTATTGTTCTCGTCTATTTTGGATATCAATCTTAGTTTTATATCTTTTCTAGTTCTCTACGTGTTAAATGTTCAACACACTAGCAATTTGGCCTGCCAGCGTATGGATTATGGAACTATCAAGTGTGTGGGATCGATAAATATGCTTCTCAGGAATTTGAGATTTTACAGTCTTTATGCTCATTGGGTTGAGTATAATATAGTAAAAAAATAGTAAATTTAAGCAATAATGTTAGGTGCTATGTGTCTGTCGAGACTATT

CTCGAGATCATATGATAATCGATATTTAATTAAATCACGTGATTACGTATAGTATACAT

GATCTAGTAACATAGATGACACCGCGCGCGATAATTTATCCTAGTTTGCGCGCTATATTTTGTTTTCTATCGCGTATTAAATGTATAATTGCGGGACTCTAATCATAAAAACCCATCTCATAAATAACGTCATGCATTACATGTTAATTATTACATGCTTAACGTAATTCAACAGAAATTATATGATAATCATCGCAAGACCGGCAACAGGATTCAATCTTAAGAAACTTTATTGCCAAATGTTTGAACGATC

TCAGAAGAACTCGTCAAGAAGGCGATAGAAGGCGATGCGCTGCGAATCGGGAGCGGCGATACCGTAAAGCACGAGGAAGCGGTCAGCCCATTCGCCGCCAAGCTCTTCAGCAATATCACGGGTAGCCAACGCTATGTCCTGATAGCGGTCCGCCACACCCAGCCGGCCACAGTCGATGAATCCAGAAAAGCGGCCATTTTCCACCATGATATTCGGCAAGCAGGCATCGCCATGGGTCACGACGAGATCATCGCCGTCGGGCATGCGCGCCTTGAGCCTGGCGAACAGTTCGGCTGGCGCGAGCCCCTGATGCTCTTCGTCCAGATCATCCTGATCGACAAGACCGGCTTCCATCCGAGTACGTGCTCGCTCGATGCGATGTTTCGCTTGGTGGTCGAATGGGCAGGTAGCCGGATCAAGCGTATGCAGCCGCCGCATTGCATCAGCCATGATGGATACTTTCTCGGCAGGAGCAAGGTGAGATGACAGGAGATCCTGCCCCGGCACTTCGCCCAATAGCAGCCAGTCCCTTCCCGCTTCAGTGACAACGTCGAGCACAGCTGCGCAAGGAACGCCCGTCGTGGCCAGCCACGATAGCCGCGCTGCCTCGTCCTGCAGTTCATTCAGGGCACCGGACAGGTCGGTCTTGACAAAAAGAACCGGGCGCCCCTGCGCTGACAGCCGGAACACGGCGGCATCAGAGCAGCCGATTGTCTGTTGTGCCCAGTCATAGCCGAATAGCCTCTCCACCCAAGCGGCCGGAGAACCTGCGTGCAATCCATCTTGTTCAATCAT

TGCAGATTATTTGGATTGAGAGTGAATATGAGACTCTAATTGGATACCGAGGGGAATTTATGGAACGTCAGTGGAGCATTTTTGACAAGAAATATTTGCTAGCTGATAGTGACCTTAGGCGACTTTTGAACGCGCAATAATGGTTTCTGACGTATGTGCTTAGCTCATTAAACTCCAGAAACCCGCGGCTGAGTGGCTCCTTCAACGTTGCGGTTCTGTCAGTTCCAAACGTAAAACGGCTTGTCCCGCGTCATCGGCGGGGGTCATAACGTGACTCCCTTAATTCTCCGCTCATGATCAGATTGTCGTTTCCCGCCTTCAGTTTAAACTATCAGTGTTGGCCGGCG

CCTAGGTTCGTACGATTCGCGAATGGTACCATATTTAAAT

AAAT

GTTTACACCACAATATATCCTGCCA

GGGCCCATTTCGAAAAAGGCCGGCC

CCAGCCAGCCAACAGCTCCCCGACCGGCAGCTCGGCACAAAATCACCACTCGATACAGGCAGCCCATCAGTCCGGGACGGCGTCAGCGGGAGAGCCGTTGTAAGGCGGCAGACTTTGCTCATGTTACCGATGCTATTCGGAAGAACGGCAACTAAGCTGCCGGGTTTGAAACACGGATGATCTCGCGGAGGGTAGCATGTTGATTGTAACGATGACAGAGCGTTGCTGCCTGTGATCAAATATCATCTCCCTCGCAGAGATCCGAATTATCAGCCTTCTTATTCATTTCTCGCTTAACCGTGACAGGCTGTCGATCTTGAGAACTATGCCGACATAATAGGAAATCGCTGGATAAAGCCGCTGAGGAAGCTGAGTGGCGCTATTTCTTTAGAAGTGAACGTTGACCATATCAACTCCCCTATCCATTGCTCACCGAATGGTACAGGTCGGGGACCCGAAGTTCCGACTGTCGGCCTGATGCATCCCCGGCTGATCGACCCC

AGATCTGGCGCCGGCCAGCGAGACGAGCAAGATTGGCCGCCGCCCGAAACGATCCGACAGCGCGCCCAGCACAGGTGCGCAGGCAAATTGCACCAACGCATACAGCGCCAGCAGAATGCCATAGTGGGCGGTGACGTCGTTCGAGTGAACCAGATCGCGCAGGAGGCCCGGCAGCACCGGCATAATCAGGCCGATGCCGACAGCGTCGAGCGCGACAGTGCTCAGAATTACGATCAGGGGTATGTTGGGTTTCACGTCTGGCCTCCGGACCAGCCTCCGCTGGTCCGATTGAACGCGCGGATTCTTTATCACTGATAAGTTGGTGGACATATTATGTTTATCAGTGATAAAGTGTCAAGCATGACAAAGTTGCAGCCGAATACAGTGATCCGTGCCGCCCTGGACCTGTTGAACGAGGTCGGCGTAGACGGTCTGACGACACGCAAACTGGCGGAACGGTTGGGGGTTCAGCAGCCGGCGCTTTACTGGCACTTCAGGAACAAGCGGGCGCTGCTCGACGCACTGGCCGAAGCCATGCTGGCGGAGAATCATACGCATTCGGTGCCGAGAGCCGACGACGACTGGCGCTCATTTCTGATCGGGAATGCCCGCAGCTTCAGGCAGGCGCTGCTCGCCTACCGCGATGGCGCGCGCATCCATGCCGGCACGCGACCGGGCGCACCGCAGATGGAAACGGCCGACGCGCAGCTTCGCTTCCTCTGCGAGGCGGGTTTTTCGGCCGGGGACGCCGTCAATGCGCTGATGACAATCAGCTACTTCACTGTTGGGGCCGTGCTTGAGGAGCAGGCCGGCGACAGCGATGCCGGCGAGCGCGGCGGCACCGTTGAACAGGCTCCGCTCTCGCCGCTGTTGCGGGCCGCGATAGACGCCTTCGACGAAGCCGGTCCGGACGCAGCGTTCGAGCAGGGACTCGCGGTGATTGTCGATGGATTGGCGAAAAGGAGGCTCGTTGTCAGGAACGTTGAAGGACCGAGAAAGGGTGACGATTGATCAGGACCGCTGCCGGAGCGCAACCCACTCACTACAGCAGAGCCATGTAGACAACATCCCCTCCCCCTTTCCACCGCGTCAGACGCCCGTAGCAGCCCGCTACGGGCTTTTTCATGCCCTGCCCTAGCGTCCAAGCCTCACGGCCGCGCTCGGCCTCTCTGGCGGCCTTCTGGCGCTCTTCCGCTTCCTCGCTCACTGACTCGCTGCGCTCGGTCGTTCGGCTGCGGCGAGCGGTATCAGCTCACTCAAAGGCGGTAATACGGTTATCCACAGAATCAGGGGATAACGCAGGAAAGAACATGTGAGCAAAAGGCCAGCAAAAGGCCAGGAACCGTAAAAAGGCCGCGTTGCTGGCGTTTTTCCATAGGCTCCGCCCCCCTGACGAGCATCACAAAAATCGACGCTCAAGTCAGAGGTGGCGAAACCCGACAGGACTATAAAGATACCAGGCGTTTCCCCCTGGAAGCTCCCTCGTGCGCTCTCCTGTTCCGACCCTGCCGCTTACCGGATACCTGTCCGCCTTTCTCCCTTCGGGAAGCGTGGCGCTTTTCCGCTGCATAACCCTGCTTCGGGGTCATTATAGCGATTTTTTCGGTATATCCATCCTTTTTCGCACGATATACAGGATTTTGCCAAAGGGTTCGTGTAGACTTTCCTTGGTGTATCCAACGGCGTCAGCCGGGCAGGATAGGTGAAGTAGGCCCACCCGCGAGCGGGTGTTCCTTCTTCACTGTCCCTTATTCGCACCTGGCGGTGCTCAACGGGAATCCTGCTCTGCGAGGCTGGCCGGCTACCGCCGGCGTAACAGATGAGGGCAAGCGGATGGCTGATGAAACCAAGCCAACCAGGAAGGGCAGCCCACCTATCAAGGTGTACTGCCTTCCAGACGAACGAAGAGCGATTGAGGAAAAGGCGGCGGCGGCCGGCATGAGCCTGTCGGCCTACCTGCTGGCCGTCGGCCAGGGCTACAAAATCACGGGCGTCGTGGACTATGAGCACGTCCGCGAGCTGGCCCGCATCAATGGCGACCTGGGCCGCCTGGGCGGCCTGCTGAAACTCTGGCTCACCGACGACCCGCGCACGGCGCGGTTCGGTGATGCCACGATCCTCGCCCTGCTGGCGAAGATCGAAGAGAAGCAGGACGAGCTTGGCAAGGTCATGATGGGCGTGGTCCGCCCGAGGGCAGAGCCATGACTTTTTTAGCCGCTAAAACGGCCGGGGGGTGCGCGTGATTGCCAAGCACGTCCCCATGCGCTCCATCAAGAAGAGCGACTTCGCGGAGCTGGTGAAGTACATCACCGACGAGCAAGGCAAGACCGAGCGCCTTTGCGACGCTCA


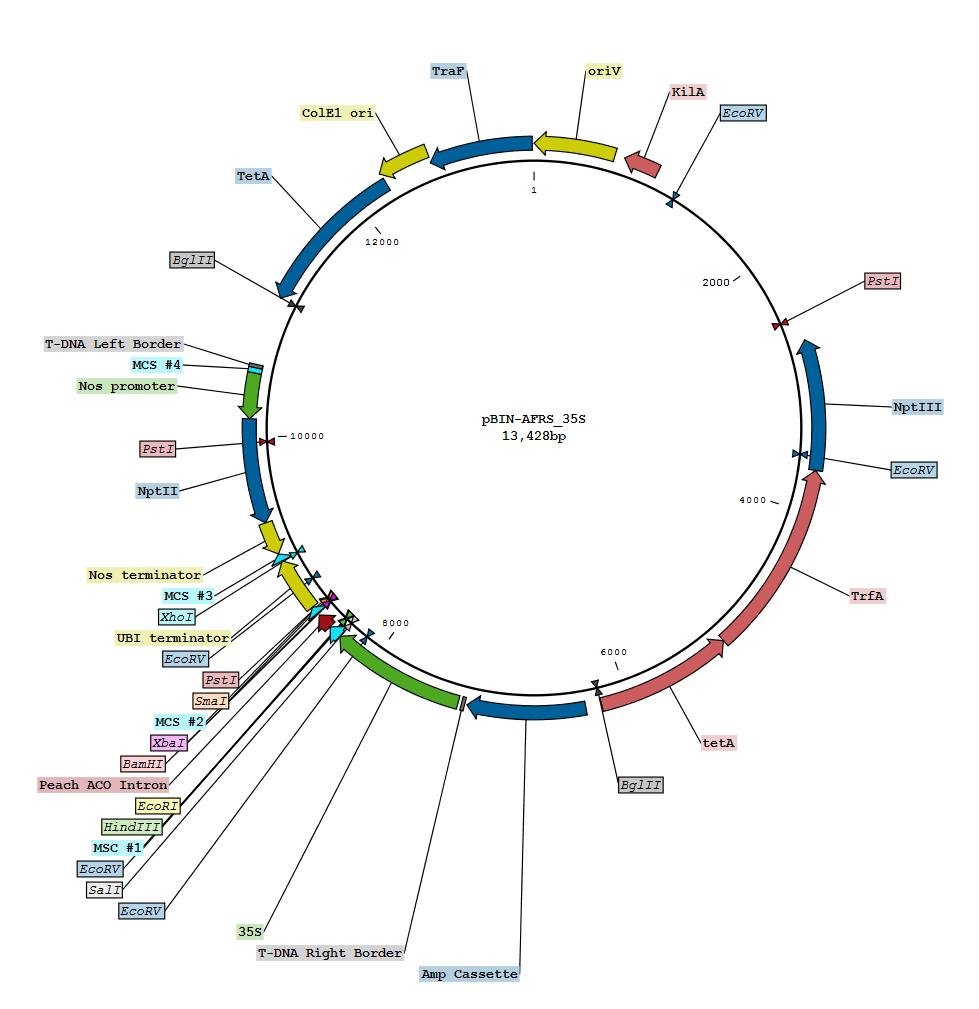

Supplement: Supplementary file 3 — Datafile S1 [file 41438_2018_34_MOESM3_ESM.docx]
